# Genetic basis for prediction of non-responders to dietary plant sterol intervention (GenePredict-PS): a study protocol for a double-blind, placebo-controlled, randomized two-period crossover study

**DOI:** 10.1186/s13063-020-04364-5

**Published:** 2020-06-01

**Authors:** Maryam Shamloo, Matthew J. Granger, Elke A. Trautwein, James D. House, Dylan MacKay

**Affiliations:** 1 Department of Food and Human Nutritional Sciences, Faculty of Agriculture and Food Sciences, University of Manitoba, Winnipeg, MB Canada; 2 Unilever R & D Vlaardingen, Vlaardingen, The Netherlands; 3 Department of Community Health Sciences, University of Manitoba, Winnipeg, MB Canada

**Keywords:** Plant sterols, Cholesterol, Genetic, SNPs, Prediction

## Abstract

**Background:**

Functional food ingredients and natural health products have been demonstrated to reduce disease risk and thereby help to lower health care costs across populations at risk for chronic or degenerative diseases. However, typically a wide range of interindividual variability exists in response across individuals to nutritional and natural health product bioactives, such as plant sterols (PS). This study aims to determine and utilize information on the associations between genosets and the degree of responsiveness to dietary PS intervention, with a long-term objective of developing genetic tests to predict responses to PS.

**Methods:**

This clinical trial is designed as a double-blind, placebo controlled, randomized two-period crossover study. Sixty-four eligible participants with the specific a priori-determined single nucleotide polymorphisms (SNPs) associated with a responsiveness to PS will consume PS or a placebo treatment for two 4-week periods. The PS treatment consists of two daily single portions of margarine, each providing 1 g PS during the PS period (2.0 g/day of PS in total). The placebo will be an identical margarine containing no added PS. Low-density lipoprotein cholesterol (LDL-C) responsiveness to the controlled administration of PS will be investigated as the primary outcome, and the associations between interindividual genoset variabilities and response to PS consumption will be determined.

**Discussion:**

This research will provide further insight into whether the associations between previously identified SNPs and the response of LDL-C to PS consumption can be used in a predictive manner. It will also provide insight into the complexities of undertaking a nutrigenetic trial with prospective recruitment based on genotype.

**Trial registration:**

ClinicalTrials.gov: Identifier: NCT02765516. Registered on 6 May 2016.

## Background

Elevated blood concentrations of low density lipoprotein cholesterol (LDL-C) is an important risk factor for cardiovascular disease (CVD) [1]. Evidence suggests that the incidence of coronary heart disease (CHD) is proportionally reduced by lowering the LDL-C [2] and that CHD is the primary target when initiating lipid-lowering interventions in the current guidelines [3]. LDL-C reduction strategies include diet and lifestyle changes, pharmaceutical therapies, intestinal bypass surgery, and lipid apheresis [3].

Changes in dietary habits can play a critical role in the reduction of LDL-C concentrations; for example, supplementation with functional food ingredients such as dietary fibers and plant sterols (PS) has been demonstrated to reduce LDL-C [4] and thereby help to lower healthcare costs across populations with chronic or degenerative diseases [5]. However, a wide range of interindividual variability in responsiveness to natural health product bioactives, such as soluble fiber and PS, has been reported [6]. Understanding this existing interindividual variability in responsiveness is important for public health and for functional food manufacturers because it may help them predict which individuals might or might not receive benefits from consuming a particular functional food or natural health product.

A better understanding of the cause of such interindividual variability, especially the impact of genetics, can help to inform individuals about optimal dosing strategies and can contribute to the development of a method that can determine, before use of a product begins, whether an individual will benefit from a particular bioactive. For instance, if an individual knows that they are a non-responder to a bioactive, they then may be able to choose other therapeutic products or approaches.

A series of predictive response tests for nutritional bioactives based on genotype would represent a genomics-derived solution and would promote a better understanding of the wide range of interindividual variability in responses to nutritional bioactives.

PS are a nutritional bioactive for which such a predictive test would be helpful. The clinical efficacy of consuming added PS for lowering LDL-C is demonstrated in the vast number of clinical studies, as summarized in several meta-analyses [7–9]. The concept of a predictive responsiveness test for PS supplementation is based on our research findings of previously completed human nutrition intervention trials [10–12]. In a recent intervention trial, the response of LDL-C to PS consumption was associated with SNPs in cholesterol 7 alpha-hydroxylase (*CYP7A1, rs3808607*) and apolipoprotein E (*ApoE*, *rs7412* and *rs429358*) (Table 1) [13]. A key discovery from these trials was that combinations of these SNPs (known as genosets) were found to interact with each other to form stronger associations with the magnitude of LDL-C lowering in response to PS consumption than for each SNP alone. However, these associations were established post hoc in a trial that selected for individuals with high or low cholesterol synthesis. An a priori approach replicating these findings is required to provide evidence that these genosets could indeed be used as a predictive responsiveness test.

**Table 1:** Single nucleotide polymorphisms (SNPs) for plant sterol responsiveness testing

Gene *SNP*	Function of gene	Association with plant sterol response
**CYP7A1** *rs3808607*	The rate-limiting enzyme in the synthesis of bile acid in the classic pathway.	T/T = non-responsive G/T = responsive G/G = responsive
**ApoE** Variant	Apolipoprotein E is a glycoprotein present in human plasma; ApoE is associated with triglyceride-rich lipoproteins (chylomicrons and VLDLs) and HDL.	ε2/− = Unknown ε3/ε3 = neutral ε4/− = responsive

To the best of our knowledge, no clinical trial so far has investigated the associations between certain SNPs and/or genosets and the degree of responsiveness to a dietary PS intervention in an a priori fashion.

Therefore, the objective of this study is to determine and utilize information on the associations between SNPs and the degree of responsiveness to dietary PS intervention, with the long-term goal of developing a predictive responsiveness test.

Therefore, the primary specific hypothesis of this study after these protocol amendments are 1) the genoset formed from *CYP7A1 rs3808607T/T* and *APOE E3/3* predict nonresponse*,* and 2) *APOE isoform ε4/−* and *CYP7A1 rs3808607 G/−* will independently predict response to PS consumption in a pattern that reflects the current gene-biomarker associations outlined in Table 1. For these hypotheses, response is being defined as a reduction in the LDL-C concentrations due to plant sterol consumption.

## Methods/Design

### Study design

To formally validate whether *APOE isoform*, which is formed by *rs7412* and *rs429358,* and *CYP7A1 rs3808607* can predict responsiveness to PS consumption across the general population, the present proposal is to carry out a double-blind, placebo-controlled, randomized two-period crossover study to investigate the LDL-C responsiveness to the controlled administration of PS. The PS treatment will consist of two daily single portions of margarine, providing 1 g each of PS during the PS period (2.0 g/day of PS in total). The placebo treatment will be an identical margarine, except it will not contain any added PS. Both the PS and placebo margarine treatments will be coded by the industrial partner organization, Unilever, and provided to the research group to maintain blinding of both the researchers and participants throughout the clinical trial.

We have two original specific hypotheses. 1) *APOE isoform* and *CYP7A1 rs3808607* will independently predict the response to PS consumption in a pattern that reflects the current gene-biomarker associations as outlined in Table 1. *APOE ε4/−* will be more responsive to PS than *ε3/ε3*. The *CYP7A1 rs3808607* G allele will predict responsiveness to PS consumption in a dose-responsive fashion, with T/T predicting nonresponse. 2) The genosets formed by combinations of *APOE isoform* and *CYP7A1 rs3808607* will follow the pattern as predicted in Table 2.

**Table 2:** Original plant sterol trial genotype recruitment targets and predicted response

ApoE	CYP7A1	Predicted response	Planned recruitment
ε2/−	T/T	Nonresponder	*n* = 8
ε2/−	G/−	Responder	*n* = 8
ε3/ε3	T/T	Non-responder	*n* = 8
ε3/ε3	T/G	Responder	*n* = 8
ε3/ε3	G/G	Responder	*n* = 8
ε4/−	T/T	Responder	*n* = 8
ε4/−	T/G	Responder	*n* = 8
ε4/−	G/G	Responder	*n* = 8

Because of the amount of time spent on recruitment and the difficulty in finding participants who were eligible with rare combinations of genosets, the APOE 2 groups were removed, and other groups were combined. To maintain the study power, we increased the *n* in the other groups as described in Table 3.

**Table 3:** Amended plant sterol trial genotype recruitment targets and predicted response

ApoE	CYP7A1	Predicted response	Planned recruitment
ε3/ε3	T/T	Nonresponder	*n* = **20**
ε3/ε3	G/−	Responder	*n* = 22
ε4/−	−/−	Responder	*n* = 22

The trial will use a priori recruitment of 64 individuals (Table 3) with the specific SNPs associated with responsiveness to PS at a) the University of Manitoba’s Richardson Centre for Functional Foods and Nutraceuticals (RCFFN) and b) Seven Oaks General Hospital (SOGH) in Winnipeg, Manitoba, Canada. The present trial will therefore select individuals from the general population with specific SNPs and then test their responsiveness to PS consumption. These responsiveness characterizations will generate the required data to validate the genoset-based classifications of responders and nonresponders.

**Fig. 1:**
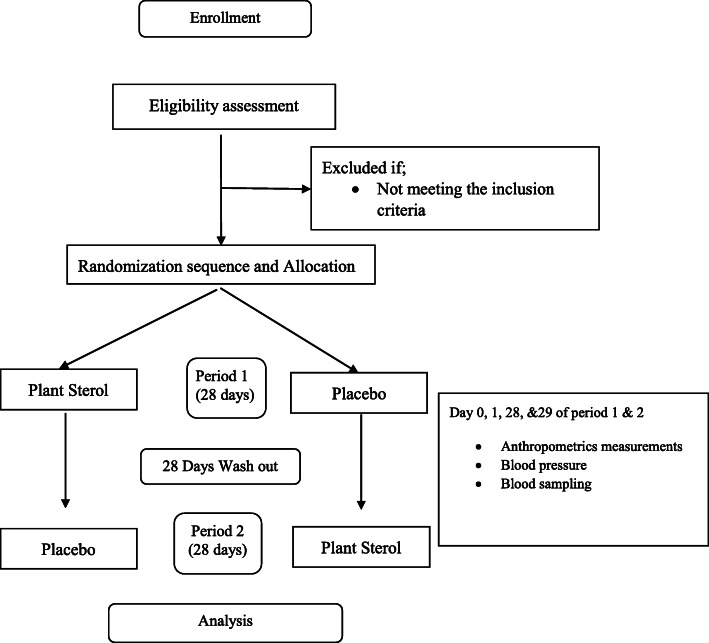
Schematic flow diagram of the trial protocol

Each treatment period will consist of 28 days, with a minimum washout of 21 days between periods. Figure 1 shows the schematic flow diagram of the trial protocol.

Participants will be required to attend breakfast at the RCFFN or Seven Oaks General Hospital (SOGH) and consume a meal containing one daily portion of margarine under supervision from Monday to Friday; the additional daily portion will be consumed with their evening meal. Participants will be given their evening and weekend margarine portions to take home for consumption. Participants will be provided diaries in which they are instructed to record when they ate the margarine in the evenings and on the weekends. During the week, participants will be required to return the empty margarine tubs on the following day to help monitor compliance, with those margarine tubs used on Saturday and Sunday being returned on Monday. The return of the empty tubs and the record of the consumption in the study diaries will be verified by clinical coordinators using a compliance checklist. Additionally, serum noncholesterol sterols, including sitosterol and campesterol, the two main PS in the margarine, will be measured to monitor compliance. Partial supervision of treatment consumption in person and the monitoring of unsupervised treatments by return of the empty container represent a compromise between participant compliance and participant burden.

Missed treatment consumption and return of margarine tubs will be recorded for each participant. Noncompliance of a participant will be defined as 1) missing supervision, 2) failing to return at least 80% of the total empty margarine tubs per treatment period, and 3) missing two consecutive measurements or blood sampling days. Noncompliant participants will be asked to leave the trial; however, they will be compensated on a prorated basis according to the duration of their involvement in the trial. Participants were asked to maintain their typical diet and physical activity levels throughout the study.

Additionally, on a weekly basis, clinical coordinators will ask participants to report any changes in diet, lifestyle (sleep), or physical activity, which may interfere with the results of the trial and any other health outcomes or symptoms they may experience during the trial. Fasting blood samples are collected from participants on 2 consecutive days at the beginning (Days 0 and 1) and at the end (Days 28 and 29) of each trial period as described in the Table 4.

**Table 4:** Schedule of enrollment, interventions, and assessments

	Study period (weeks)					
Visit #	Screening	1, 2	3, 4	4 week wash out	5, 6	7, 8
Study week		0	4 (+ 5 / -2 days)		9 (+ 5 / -2 days)	12 (+ 5 / -2 days)
Informed consent, demographic information, inclusion/exclusion criteria, and medical history	**+**					
Vital signs and anthropometric measures (body weight, hip and waist circumference, BMI, blood pressure, arterial stiffness)	**+**	**+**	**+**		**+**	**+**
Concomitant medications	**+**	**+**	**+**		**+**	**+**
Genotyping of DNA samples	**+**					
Blood lipid profile (TG, TC, LDL-C, HDL-C) and glucose	**+**	**+**	**+**		**+**	**+**
Blood sterol and sterol precursor profile		**+**	**+**		**+**	**+**
Gastrointestinal (GI) tolerability questionnaires		**+**	**+**		**+**	**+**
Treatment Dispensation		**+**			**+**	
Treatment Accountability (Participant Consumption Checklist)			**+**			**+**
treatment checklists			**+**			**+**
Adverse events			**+**			**+**
Study termination						**+**

### Study participants

The participants (64 in total) will be recruited using various established methods, including the use of flyers around the University of Manitoba and Seven Oaks General Hospital (SOGH), newspaper advertisements, direct mail advertisements within the City of Winnipeg, and digital advertisements at the Active Living Center of University of Manitoba, as well as SOGH social media, websites and newsletter advertising among 6500 members of SOGH. An internal list of previous volunteers who have expressed interest in participating in other clinical studies will also receive an advertisement. Participants will be initially screened for eligibility over the telephone by the study coordinator if they respond to an advertisement. If eligible, potential participants will be invited to the clinical research unit at the RCFFN or SOGH for an information session to introduce the research staff and provide further information about the study. Those expressing further interest will be invited to consent to and have a blood sample taken to ensure they meet all other trial criteria as listed below. Blood samples will be taken by RCFFN or SOGH phlebotomists or registered nurses. Screening blood samples are analyzed for the following: fasting lipid profiles including LDL-C, total cholesterol (TC), high density lipoprotein cholesterol (HDL-C), and triglyceride (TG) concentrations, as well as, glucose, serum creatinine, blood urea nitrogen (BUN), aspartate aminotransferase (AST), alanine aminotransferase (ALT), gamma-glutamyl transferase (GGT), serum total protein, and serum albumin; all will be measured using the automated enzymatic methods on the Cobas 311 Analyzer (Roche Diagnostics GmbH, Mannheim, Germany) or measured by Diagnostic Services Manitoba (DSM) according to their standard protocols. DNA will be extracted from the blood sample buffy coat using commercially available column-based DNA extraction kits (DNeasy Blood and Tissue Kit, QIAGEN Sciences) according to the manufacturer’s instructions. The concentration and integrity of the genomic DNA will be assessed by micro-volume spectrophotometer (NanoDrop 2000, Thermo Fisher Scientific). DNA samples will then be genotyped by TaqMan SNP genotyping assays (CYP7A1-rs3808607, assay identification (ID) C2749212120; APOE rs7412, assay ID C2749212120; APOE-rs429358, assay ID C308479320; Life Technologies) on a StepOnePlus Real-Time PCR System (Applied Biosystems; Life Technologies). Data from the screening blood sample will be used to screen participants based on the predefined inclusion and exclusion criteria.

#### Inclusion criteria

Men and women aged 18–70 years and with LDL-C concentrations of 3.0–4.9 mmol/L will be recruited into the trial. Participants must have a fasting glucose concentration of < 6.1 mmol/L. A prospective recruitment scheme based on genotype will recruit 20–22 individuals in each of the three most common combinations, also called genosets, (outlined in Table 3). This approach would leave a minimum sample size of 20 participants for each individual genotype. The prospective recruitment based on genosets of interest will require screening of 200–400 potential participants. Such lengthy screening is required to find sufficient individuals who have the rarer genosets and meet all the inclusion and exclusion criteria. Additionally, participants must be willing to fast for 10–12 h before blood sampling, abstain from alcohol for 2 days prior to blood sampling, and abstain from coffee and physical exercise for at least 12 h before measurements and blood sampling. All participants must be able and willing to give informed consent to participate in the trial prior to their inclusion.

#### Exclusion criteria

Participants will be excluded if they are consuming or have consumed in the last 3 months medications or nutritional supplements known to affect lipid metabolism (such as cholestyramine, colestipol, niacin, clofibrate, gemfibrozil, probucol, HMG-CoA reductase inhibitors (statins), methotrexate, high-dose dietary fiber supplements, or plant sterols or stanols), or have any dietary restrictions which would prevent them from consuming the trial treatments. Participants who have a BMI > 40 kg/m^2^ will be excluded. Participants must not have self-reported weight gain or loss greater than 3 kg in the past 3 months. Participants must be free of active cardiovascular disease including stroke; congestive heart failure; myocardial infarction; unstable angina pectoris; coronary artery bypass graft; percutaneous transluminal coronary angioplasty; temporal ischemic attacks; anemia; abnormal electrolytes; proteinuria; and abnormal liver, kidney, or thyroid function. Participants will be excluded if they have clinically significant biochemistry defined as: LDL-C < 3.0 mmol/L or > 4.9 mmol/L, TC > 6.2 mmol/L, fasting glucose > 6.1 mmol/ L, fasting TG > 4.52 mmol/L, AST > 100 U/L, ALT > 100 U/L, or at the investigator’s discretion, for any other clinically significant abnormalities in hematology and/or biochemistry.

Participants will be excluded if they have phytosterolemia, type 1 or type 2 diabetes, a history of cancer or malignancy in the last 5 years, or any metabolic disease, gastrointestinal disorder, or other clinically significant disease/disorder that could interfere with the results of the study or the safety of the participant. Participants will be excluded if they are smokers, tobacco/snuff/nicotine users, recreational drug users, or if they consume more than 14 alcoholic beverages a week. Participants who are pregnant or plan to become pregnant during the trial period will be excluded. Lactating women will also be excluded. Patients with unstable or serious illness, for example, dementia, terminal illness, recent bereavement, or recent significant medical diagnosis, will also be excluded. Employees of Unilever, Nutritional Fundamentals of Health (NFH) and the research institutes conducting the research will not be allowed to participate in the study.

### Randomization

Eligible participants will be randomly allocated to two groups: the PS treatment group or the placebo group for the first period, and then, participants will switch treatments for the second period after the washout between periods. Randomization will be done by an assistant outside of the research team using a block randomization method through sealed envelopes with stratification by sex and genoset. Randomization in blocks of eight and four, each with equal numbers of treatment orders will be used. This blocking is being done to minimize imbalances in treatment orders within each genoset group or by sex. Administration of the intervention will be conducted in a double-blind manner. Single portion tubs of PS treatment and placebo margarine are being created for this study by Unilever and are being delivered to the research team in identical packages labeled either A or B.

### Remuneration

Study participants will receive up to a total of CAD $400 (i.e., $200/period × 2 periods) for study completion. This amount will be divided into two portions. Participants will receive $200 after the completion of period 1 and another $200 after the completion of period 2. If a participant withdraws early from the study, the participant will receive an appropriate prorated fraction of this amount.

## Outcome measures

### Primary outcome

Serum LDL-C concentration and its change in response to PS consumption is considered the primary outcome of this trial. This outcome was chosen to measure plant sterol response in terms of lowering of the LDL-C concentration between the placebo and plant sterol consumption period. Blood samples (20 mL) will be collected on days 0, 1, 28, and 29 of the intervention period. The serum lipid profile (TC, LDL-C, HDL-C, and TG) will be measured using the Cobas 311 Analyzer (Roche Diagnostics GmbH, Mannheim, Germany). The average value for days 0 and 1 will be used as the baseline, and the average of days 28 and 29 will be the endpoint values.

### Secondary outcomes

At baseline and at the end of the two intervention periods, anthropometric measurements, including body weight, BMI, hip and waist circumference, and blood pressure, will be taken. Blood pressure will be measured in an office setting on. Days 0, 1, 28, and 29 of each treatment period. Participants will be asked to rest 10 min prior to having their blood pressure taken, in case they had rushed into the setting. This measurement will take place in a quiet room while the participant is in a seated position, with the arm rested on an armrest at heart level. Participants will be advised to rest quietly throughout the measurements. Blood pressure measurement will be performed four times at 2-min intervals. Gastrointestinal tolerability questionnaires will be completed by the participants at the beginning and the end of each intervention period. The 10-year CVD risk score will be calculated for each participant during each intervention period utilizing the ACC/AHA Cholesterol Guideline risk calculator. Upon completing the trial, participants will be asked to complete a questionnaire that asks them whether they think they know which treatment they received during each treatment period. This information will be used to verify participant blinding.

Fasting serum glucose will be measured with the Cobas 311 Analyzer (Roche Diagnostics GmbH, Mannheim, Germany). Plasma samples will be used to quantify concentrations of blood sterols and sterol precursors (noncholesterol sterols, NCS) according to a previously established method [11]. Authenticated internal standards will be added to plasma samples, which will then be saponified with methanolic KOH solution. Sterols will then be extracted twice with petroleum ether. Extracted sterols will be derivatized using a trimethylsilylation (TMS) procedure. The TMS-derivatized samples and sterol analysis will be carried out by gas chromatography with flame ionization detection. Campesterol, sitosterol, campestanol, sitostanol, and cholestanol, as well as lanosterol, desmosterol, and lathosterol will be measured.

Fractional cholesterol synthesis will be measured by deuterium incorporation according to previously established procedures [10, 13, 14]. Twenty-four hours before the end of each treatment period participants will be asked to consume deuterium water (D_2_O) given at a dose of 0.7 g/kg body water (estimated at 60% of total body weight). D_2_O is a stable isotopic tracer and poses no radiation hazard and can be safely administered to human participants. D_2_O water will be administered orally. A fasted blood sample will be taken at baseline and on day 28 prior to isotope administration, in addition to the fasting samples taken on day 29. The change in deuterium enrichment within red blood cell free cholesterol will be determined as an index of cholesterol synthesis over days 28 and 29.

## Sample size calculation and statistical analysis

The sample size, with a minimum of 20 participants for each individual genotype and *n* = 20–22 for each individual genoset, is based on previous work performed by this research group [13]. A power calculation was performed using PROC POWER SAS Institute (version 9.4) using the paired means statement to model the AB/BA crossover design (corr = 0.75, a = 0.05 and b = 0.80); based on an average reduction in LDL-C of 0.34 mmol/L resulting from PS consumption according to the meta-analysis findings of Demonty et al. [8], the standard deviations in LDL-C for placebo (0.67 mmol/L) and PS (0.7 mmol/L) from the MacKay et al. [11] and a correlation in LDL-C values of 0.75, which was an estimate based on variability in LDL-C concentrations over time from MacKay et al. [11] and on a within-persons correlation in cholesterol response to plant sterols [15]. From this power calculation, we determined that 18 participants would be needed for each genoset to detect a response, in the form of a significant reduction in LDL-C from placebo to plant sterol consumption period, in the group. Our objective was to test if each genoset would respond, with the hypothesis that the *CYP7A1 rs3808607T/T* and *APOE E3/3* genoset would not respond to plant sterol consumption with LDL-C lowering.

Given the crossover design, the study outcomes measures will be analyzed in a per-protocol population where only participants who received both treatment and placebo are included. The effects of treatment, comparing the endpoint values of the treatment and placebo periods, will be analyzed by the SAS MIXED procedure. Sequence and sex will be included in the model as fixed factors, while participants will be included as a random and repeated factor. Genoset and treatment by genoset will be included as fixed factors to assess the impact of the genoset on the treatment. The impact of the individual genotypes will also be investigated individually. Significant treatment-by-genoset or treatment-by-genotype effects will be examined by the SAS SLICE function, with Bonferroni correction for the number of slices. Treatment effect sizes by genoset or genotype, from significant interactions, will be compared by t test or ANOVA using the difference in mixed-model least squares means summary statistics for the treatment effect slices, with Tukey-Kramer adjustment for multiple comparisons [13].

## Discussion

In a recent clinical trial by our group, the response of LDL-C to PS consumption was associated with SNPs in cholesterol 7 alpha-hydroxylase (*CYP7A1, rs3808607*) and apolipoprotein E (*ApoE*, *rs7412* and *rs429358*) [13]. This ongoing GenePredict-PS clinical trial is investigating if this previous association identified between the SNPs and the LDL-C response to PS consumption can be used in a predictive manner. Individuals with genosets that fail to reach significant reductions in plasma LDL-C levels in response to PS consumption will be classified as nonresponders, whereas those who do exhibit LDL-C lowering will be classified as responders (see predicted response in Table 3). Individuals who are classified by the genosets as responders could be advised to consider PS-added products for lowering their elevated blood total and especially LDL-cholesterol, while non-responders could be either recommended to modify the dose of PS or use other pharmaceutical or natural health products that may lower cholesterol through other pathways. Very few studies in nutrigenetics and nutrition have yet to explore recruitment of participants a priori based on genotype, let alone based on combinations of genotypes (genosets). Previously, the impact of *rs1801133,* a variant in the methylenetetrahydrofolate reductase (MTHFR) gene, on riboflavin supplementation and blood pressure has been explored [16]. In that trial, Wilson et al. were able to use an available population of 1427 patients with hypertension from which they were able to recruit individuals based on genotype. The strategy of recruiting directly from a previous genotyped population can be highly recommend given the difficulty that the current trial has faced with de novo recruitment from the general public. Recruitment of previously genotyped individuals may be the most suitable way a priori nutrigenetic studies can be carried out in a suitable fashion, especially if the studies will be recruiting based on genosets or polygenic risk scores [17].

### Trial status

This trial is ongoing and has been recruiting since July 2016. The trial is expected to continue until approximately June 2020. The current protocol number and date is version 5 and 20 July 2018, respectively. 

## Data Availability

The de-identified datasets, that will be used and/or analyzed during the current study will be available from the corresponding author on reasonable request.

## Abbreviations

ALT: alanine aminotransferase
AST: aspartate aminotransferase
BREB: Biomedical Research Ethics Board
BUN: blood urea nitrogen
BMI: body mass index
CVD: cardiovascular disease
*CYP7A1*: cholesterol 7 alpha-hydroxylase
DNA: deoxyribonucleic acid
D_2_O: deuterium water
DSM: Diagnostic Services Manitoba
EMSA: electrophoretic mobility shift assay
GGT: gamma-glutamyl transferase
HDL: high-density lipoprotein
LDL-C: low-density lipoprotein cholesterol
PS: plant sterols
RCFFN: Richardson Centre for Functional Foods and Nutraceuticals
SAS: Statistical Analysis Software
SOGH: Seven Oaks General Hospital
SNPs: single-nucleotide polymorphisms
TC: total cholesterol
TG: triglycerides
TMS: trimethylsilylation
VLDL: very low-density lipoprotein
